# Quarterly Summary

**Published:** 1856-10

**Authors:** 


### QUARTERLY SUMMARY.

1.—*Cylindrically Prepared Foil.*—Dr. White, one of the editors of the Dental News Letter, in the April No. of that journal, gives the manner in which Dr. J. S. Clark prepares his foil preparatory to introducing it into the cavity of the tooth it is designed to be condensed. "He cuts the foil into strips, wider or narrower as may be required, and then folds it upon itself as a surgeon makes a compress, or as a bolt of cloth is folded." "He," Dr. Clark, "considers this preferable to rolling it on a narrow watch spring, as we have been in the habit of doing for years, in making flat pellets, on account of its allowing the air to escape from between the folds during the process of condensation." Dr. White then proceeds to state the manner in which he prepares it, which is by using a cushion—and by placing a blade of scissors in the middle of the strip to be folded and pressing gently upon the cushion—the edges of the strip are raised up, as the middle of the strip is depressed, which keeps the operation to bring the edges together, and continues in this manner folding the strip until it is of the desired breadth, making some wider or narrower according to the depth of the cavity to be filled; the folded strips are then rolled into cylinders by means of a watchmaker's broach, a little larger than a cambric needle; this instrument must be square, or five or six sided, to present edges to hold on to the strip while rolling." The end of the strip to be rolled, is taken between the thumb and forefinger of the left hand, and the point of the instrument placed on the end of the strip and pressed hardly upon it, and rotated at the same time to catch and hold the strip while rolling it." The cylinder may be made harder or looser, as may be desired, as the strip is allowed to escape from between the thumb and finger between which it is held.

Dr. White remarks, that a leaf of No. 4 gold, cut into three portions, makes convenient sized strips when folded, or No. 6 gold cut into four parts, and that if the strips, when folded, should be too heavy it will require too much effort to bend the strip, and that the needle upon which it is folded will tear the inner folds and its hold upon the strip will be loosened. These cylinders may be of all sizes, between that of a No. 8 needle to that of a common quill. The lighter leaves make better cylinders than the heavy ones.

In placing these cylinders in a cavity, a large and loosely rolled one is placed in first, if a smaller one cannot be retained in its place, and then this cylinder may be pierced with a round pointed instrument and a smaller cylinder introduced into it, and so on until the cavity is filled, when the whole mass may be pressed well and filed off and polished.

Dr. White regards this method as invaluable in crown fillings.

The Dr. has found that the ends of the cylinders excite considerable irritation when they come in contact with sensitive dentine—which can be obviated, as he remarks, by placing over the sensitive part of the cavity a thin layer of folded gold before introducing the cylinders.

He remarks, that a plug of this kind is more readily polished and is not so liable to scale off in masticating as one which is made by using the gold in a coil.

2.—*Hypertrophy of the Gums.*—In the April No. (1856,) of the Dental Register of the West, a case of hypertrophy of the gums, the subject a patient of Dr. Gross, is reported by Dr. Goddard, dentist, of Louisville, Ky.

The case, from the description and cuts which accompany it, has points in common with a case of the kind published in an early number of the Journal, written by Dr. Koecker. The subject, which Dr. Goddard speaks of, was a lad aged about 10 years, of whose mouth the Dr. succeeded in taking an impression, the difficulties of which, were almost insurmountable. On looking at him, he presented the appearance as if his tongue were protruding beyond the jaws; but which upon more careful inspection, was found to proceed from the affected gums, which, projecting upwards and outwards, forced the upper lip against the nostrils, and greatly impeded respiration.

Dr. Gross remarks, that judging from the history of this case, there is great probability that it had existed from the birth of the patient. About the time the mother's attention was first called to it an abundant flow of saliva commenced, so irritating in its character as to erode the chin and other parts with which it came in contact. After several years this secretion diminished gradually in quantity and in its acrid quality. "Almost simultaneously with the enlargement of the gums was observed an enlargement of the tonsils, leading to embarrassment of breathing, which has remained up to the present time." Both affections gradually increased, the general health being good. "About the fifteenth month the two middle incisors of the lower jaw appeared, and after sometime the corresponding teeth above. The gum, however, was already so large as to conceal nearly the whole of the organs," after which the inferior deciduous cuspid and first molar of the right side issued, and these were the only teeth that were ever visible, prior to the operation performed. Dr. Gross then gives the condition in which the boy was found by him, when first consulted upon the case. "His body was short but thick set, his eyes black, the pupils dilated, and the lids fringed with long lashes, the hair dark, the face pale and pasty, the head rather ill-shaped, and the abdomen preternaturally prominent. The extremities were cold, the bowels regular, and the appetite good, the respiration was embarrassed, and performed with a kind of croaking sound. At night he snored very loud, and often waked up struggling for breath, the voice being muffled, and the articulation indistinct. His pulse was natural, his strength was rather impaired, and his muscles were thin and flabby.

"Projecting from the anterior part of the upper jaw was a tumor, of a pale color, inelastic, perfectly insensible, and of firm consistence, presenting very much the appearance of the snout of a hog, as represented in the sketch. It stood off very obliquely, and received but a very partial covering from the corresponding lip. It was rough on the surface, and was about an inch and a quarter in its antero-posterior diameter, its length having been about one inch and a half. At its free margin, which was quite irregular, was seen the tip of the left central incisor. Extending back from this tumor on each side, the whole length of the jaw, was the enlarged gum, forming a thick, broad ridge, completely embedding the teeth. At several points, particularly behind the morbid growth, was more than nine lines in width; in front and at the middle it was less. It was of a more florid color than the main tumor, but of about the same degree of consistence. Opposite the site of the bicuspid teeth, on each side, it exhibited a remarkably granulated appearance, the excrescences having a pedunculated form, and being folded upon each other. Projecting toward the roof of the mouth, it greatly encroached upon this cavity, lessening its capacity, and thus interfering with its functions, as well as with speech and respiration.

"The lower gum was in the same condition as the upper, equally hard and insensible, but less developed. It was of a bluish florid complexion, and larger in front and behind than at the intermediate points; its free surface was uneven and so prominent as to hide all the teeth, except the central incisors, the point of the right cuspid, and the cusps of each deciduous and first permanent molars.

"On looking into the fauces both tonsils were found to be much enlarged, the left, however, more than the right, and the arches of the palate were in a state of chronic inflammation. The uvula was also somewhat hypertrophied. As the respiration was much embarrassed by the state of the tonsils, I excised the redundant portion of the left of these organs as a prelimiuary step to the treatment of the other and more important affection.

"Various plans of treatment had been employed for the relief of the boy before he came under my charge, but all without advantage. Judging from the long continuance of the disease, and the great hardness of the parts, I became soon satisfied that the only remedy that held out any prospect of success was excision. Not knowing what might be the degree of vascularity of the morbid growth, I determined to attack the lower one first, removing as much at one sitting as the circumstances of the case would admit of. The patient being seated upon a chair, with the head supported against the breast of an assistant, I pared away the gum closely from the jaw and teeth, first in front and then behind, using for this purpose the knife, gum-lancet, or chisel, as appeared to be most expedient. About five ounces of blood, principally of a venous character, were lost during the operation. Behind, the excision was not very perfect, owing to the want of suitable instruments, to scrape away the thickened and callous structure. No pain was experienced during the operation, and it is therefore reasonable to suppose that the hypertrophied gum was perfectly insensible. Two of the temporary teeth were removed, the permanent set being found as well developed, or nearly so, as usual in children at this age. They were of a beautiful white color, and firmly rooted in their sockets.

On the fifth of January, three days after the above operation, the boy was taken before the medical class of the University of Louisville, with a view to the removal of the upper gum. Dr. Goddard, he eminent dentist, on this as on the former occasion, gave me his valuable assistance. By means of a chisel, slightly curved, and not very sharp, the greater portion of the morbid mass was easily pared off in front, exposing the permanent incisors, the two central of which were found to be fully developed, while the lateral incisors were only about one-third grown. In excising the gum from the posterior surface of the jaw bone in front, the anterior palatine artery of the right side was unavoidably laid open, and bled considerably before the flow could be arrested. The larger pieces of the gum being thus removed, Dr. Goddard cut and scraped off the remainder by means of scaling instruments, such as those used by dentists in removing tartar from the teeth, and which were found to be admirably adapted to the object. The proceeding was necessaily tedious on account of the frequent spitting of the patient, and was attended with the loss of at least six ounces of blood. Indeed, the lad became quite pale from this cause. No pain was experienced. One of the deciduous teeth was forced out during the operation. The lad now has in his upper jaw, the two central incisors and first molars fully developed, the lateral incisors about one-third grown, the cusps of the second molars just visible, and the temporary cuspids and second temporary molars.

"In the lower jaw are the four incisors and first permanent molars. The left second molar about one-fourth grown, and the other molar just emerging, with the right temporary cuspid and left temporary molar.

"Finally, a third operation was performed on the 17th of January, mainly by Dr. Goddard, consisted in the removal of remnants of gum between and around the teeth. The instruments employed on the occasion were the same as on the former. By means of these, every vestige of the morbid growth was cut and scraped away. The operation was again tedious and somewhat bloody, but not as much so as before. It was followed by great soreness of the whole mouth, by inability to masticate food, and even by considerable difficulty of deglutition, continuing for nearly a whole week. In consequence of these occurrences free use was obliged to be made of anodynes, especially at night. Since the last two operations the boy has been taking sulphate of iron and quinine, to improve his general health and the condition of the blood, which, as evidenced by the pallor of his countenance, has been much impoverished by the repeated losses sustained in the excision of the gums. He left Louisville on the 4th of March, still rather pale and feeble, but in fine spirits and with a good appetite. A few days before his departure I excised a portion of the right tonsil, the left, as already stated, having been removed a few days after his arrival in town. His breathing is too loud, though he no longer snores and perspires during sleep. Orders were given to keep up the steady use of his tonics, to make him exercise freely in the open air, and to put him upon a plain but nutritious diet. The tonsils and arches of the palate are to be mopped every fourth day with a pretty strong solution of nitrate of silver. The teeth are covered up to their necks with healthy gum; and thus far, there is not the slightest appearance of a return of the morbid growth.

"On closing the jaws so as to bring the grinders in contact, a space is found to exist between the front teeth, three quarters of an inch in extent. The upper lip is gradually regaining its proper position, and the contour of the face is greatly improved."

